# Alterations in the leaf lipidome of *Brassica carinata* under high-temperature stress

**DOI:** 10.1186/s12870-021-03189-x

**Published:** 2021-09-06

**Authors:** Zolian Zoong Lwe, Saroj Sah, Leelawatti Persaud, Jiaxu Li, Wei Gao, K. Raja Reddy, Sruthi Narayanan

**Affiliations:** 1 Department of Plant and Environmental Sciences, Clemson University, Clemson, SC 29634 USA; 2 Department of Biochemistry and Molecular Biophysics, Kansas State University, Manhattan, KS 66506 USA; 3 Department of Biochemistry, Molecular Biology, Entomology and Plant Pathology, Mississippi State University, Starkville, MS 39762 USA; 4 Plant and Soil Sciences, Mississippi State University, Starkville, MS 39762 USA; 5 USDA UVB Monitoring and Research Program, Natural Resource Ecology Laboratory, Department of Ecosystem Science and Sustainability, Colorado State University, Fort Collins, CO 80523 USA

**Keywords:** *Brassica carinata*, Lipids, High-temperature stress, Leaf lipidome, Lipid remodeling, Unsaturation index, Lipid channeling

## Abstract

**Background:**

*Brassica carinata* (A) Braun has recently gained increased attention across the world as a sustainable biofuel crop. *B. carinata* is grown as a summer crop in many regions where high temperature is a significant stress during the growing season. However, little research has been conducted to understand the mechanisms through which this crop responds to high temperatures. Understanding traits that improve the high-temperature adaption of this crop is essential for developing heat-tolerant varieties. This study investigated lipid remodeling in *B. carinata* in response to high-temperature stress. A commercial cultivar, Avanza 641, was grown under sunlit-controlled environmental conditions in Soil-Plant-Atmosphere-Research (SPAR) chambers under optimal temperature (OT; 23/15°C) conditions. At eight days after sowing, plants were exposed to one of the three temperature treatments [OT, high-temperature treatment-1 (HT-1; 33/25°C), and high-temperature treatment-2 (HT-2; 38/30°C)]. The temperature treatment period lasted until the final harvest at 84 days after sowing. Leaf samples were collected at 74 days after sowing to profile lipids using electrospray-ionization triple quadrupole mass spectrometry.

**Results:**

Temperature treatment significantly affected the growth and development of Avanza 641. Both high-temperature treatments caused alterations in the leaf lipidome. The alterations were primarily manifested in terms of decreases in unsaturation levels of membrane lipids, which was a cumulative effect of lipid remodeling. The decline in unsaturation index was driven by (a) decreases in lipids that contain the highly unsaturated linolenic (18:3) acid and (b) increases in lipids containing less unsaturated fatty acids such as oleic (18:1) and linoleic (18:2) acids and/or saturated fatty acids such as palmitic (16:0) acid. A third mechanism that likely contributed to lowering unsaturation levels, particularly for chloroplast membrane lipids, is a shift toward lipids made by the eukaryotic pathway and the channeling of eukaryotic pathway-derived glycerolipids that are composed of less unsaturated fatty acids into chloroplasts.

**Conclusions:**

The lipid alterations appear to be acclimation mechanisms to maintain optimal membrane fluidity under high-temperature conditions. The lipid-related mechanisms contributing to heat stress response as identified in this study could be utilized to develop biomarkers for heat tolerance and ultimately heat-tolerant varieties.

**Supplementary Information:**

The online version contains supplementary material available at 10.1186/s12870-021-03189-x.

## Background

*Brassica carinata* A. Braun, commonly known as Ethiopian mustard, Abyssinian mustard, or carinata, is an emerging oilseed crop in North America, South America, Europe, and Australia. An essential feature of a biofuel crop is its minimal impact on land-use changes, i.e., displacing land from food and feed crops. Carinata is an off-season crop, rotation crop, and marginal-land crop that offers a potential option for a sustainable biofuel crop [1–7]. Carinata’s high content of erucic acid (40–45 %) not only improves its suitability as a biofuel crop [2, 8, 9] but also enhances its value for industrial applications such as manufacturing of plastics, lubricants, paints, leather tanning, soaps, and cosmetics [10, 11]. Furthermore, studies have reported that carinata is tolerant to abiotic stresses such as drought and cold and resistant to multiple insect pests and diseases [12–15]. Its ability to reduce summer weed species’ seed banks makes it suitable for integrated pest management in crop production systems [7, 16]. Carinata also has potential as a feed crop (due to its high protein and low fiber content [17–19]) and biofumigant (varieties with high glucosinolate content [20]). There have been concerted efforts both from public and private entities in the last few years to establish carinata as a sustainable biofuel crop in the United States [1, 16, 21–24].

Carinata is grown as a summer crop in many regions where high temperature is expected to be a significant stress during the growing season. Limited information is available on carinata’s response to high temperatures. A recent study investigated the effect of high temperature stress on carinata using 12 genotypes [25]. The same study identified that the shoot, root, and physiological traits of carinata are significantly affected by high temperature stress (27/19ºC) during germination and early growth. However, the underlying mechanisms that drive the high-temperature sensitivity are unclear [25]. To develop high-temperature tolerant varieties, it is essential to understand the mechanisms that will enable this species to adapt to high temperatures.

Previous research on other species has demonstrated that alterations in lipid metabolism significantly affect a plant’s ability to acclimate to high temperature stress [26–32]. Lipids and proteins are the major constituents of biological membranes. The dynamic nature of membrane lipid composition is essential to maintaining cellular and ultimately whole-plant homeostasis in response to fluctuations in growth temperature [33]. Multiple lipid classes have been proposed to function in stress signaling or adaptation mechanisms [29, 34–38]. It was recently found in another *Brassica* species (*Brassica napus* L., oilseed rape) that high night temperature leads to overexpression of genes involved in fatty acid catabolism, which results in up-regulated gibberellin signaling during the night-time [39]. Additionally, recent reports in other species demonstrate that heat tolerant and susceptible genotypes differ in the heat-induced changes in their lipidome. These changes have the potential as biomarkers for selecting heat-tolerant genotypes [29, 30, 32].

In the present study, we employed automated direct infusion electrospray ionization-triple quadrupole mass spectrometry (ESI-MS/MS) to investigate the heat-induced changes in the leaf lipidome of *B. carinata*. The objective was to determine the lipid-related mechanisms associated with high-temperature stress response in this species.

## Results

### High-temperature stress affects growth traits

**Fig. 1:**
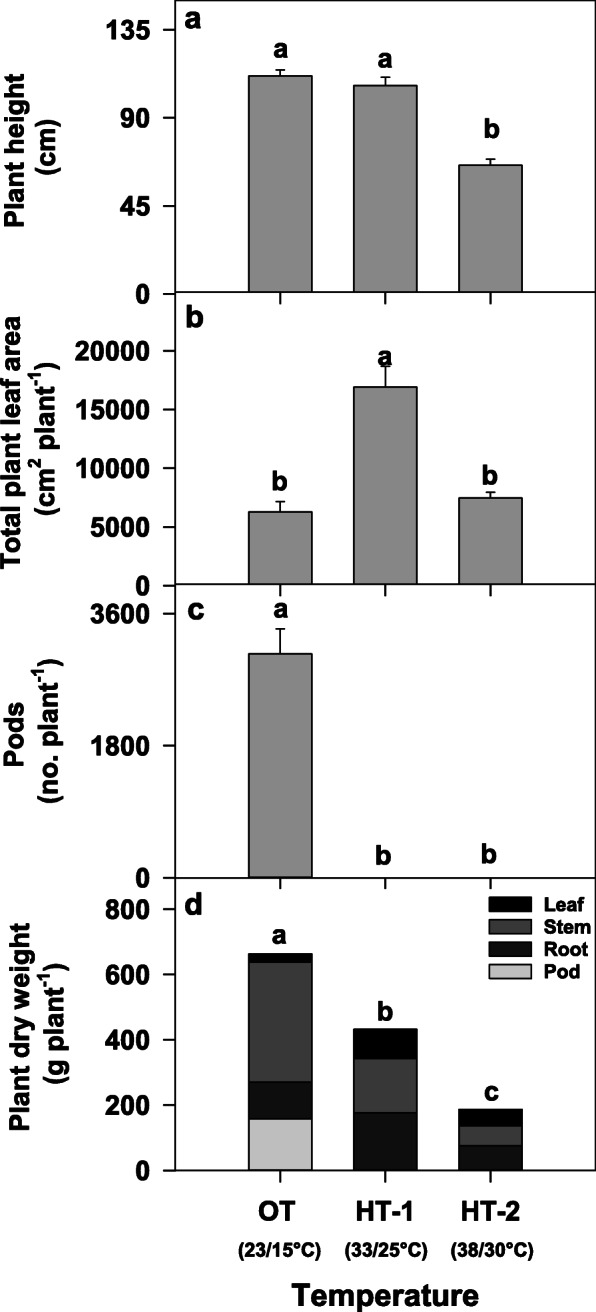
Effects of temperature on plant height (**a**), leaf area (**b**), pod number (**c**), and total dry weight (**d**) of the *Brassica carinata* cultivar Avanza 641. Total dry weight includes dry weights of leaves, stems, pods, and roots. No plants produced pods at HT-1 and HT-2. Bars represent least-squares means and error bars represent the corresponding standard errors. Least-squares means with different letters are significantly different according to Fisher’s least significant difference test at α = 0.05. OT, optimal day/night temperatures; HT-1, high temperature treatment-1; HT-2, high temperature treatment-2

Temperature treatment significantly affected the growth and development of Avanza 641 (Fig. 1). The days required to produce first bolting (inflorescence appearance) and flowering were 41.7 ± 0.33 and 47.9 ± 0.35, respectively, at the optimum temperature of 23/15°C. Plants grown at high temperatures (HT-1, 33/25 and HT-2, 38/30°C) did not bolt or produce flowers during the 84-day period showing complete reproductive failure. Plants produced 3052 ± 342 pods plant^− 1^ at OT and none at HT-1 and HT-2. The leaf area of plants grown at HT-1 was greater (+ 169 %) than that at OT. The total plant dry weight, on the other hand, was significantly greater at OT and showed lower values at HT-1 (-35 %) and HT-2 (-72 %).

### Leaf lipids were profiled and quantified with electrospray ionization-triple quadrupole mass spectrometry (ESI-MS/MS)

The ESI-MS/MS approach quantified 105 lipid analytes in the leaves of Avanza 641 (Supplementary Table S1, Additional file 1). These lipids included plastidic lipids [DGDG, digalactosyldiacylglycerol; MGDG, monogalactosyldiacylglycerol; and PG, phosphatidylglycerol], extraplastidic lipids [PC, phosphatidylcholine; PE, phosphatidylethanolamine; PI, phosphatidylinositol; PS, phosphatidylserine, LPC, lysophosphatidylcholine; and LPE, lysophosphatidylethanolamine], and phosphatidic acid (PA). Analysis of variance results revealed significant changes in the composition of the leaf lipidome under high-temperature stress (Supplementary Table S2, Additional file 1).

**Fig. 2:**
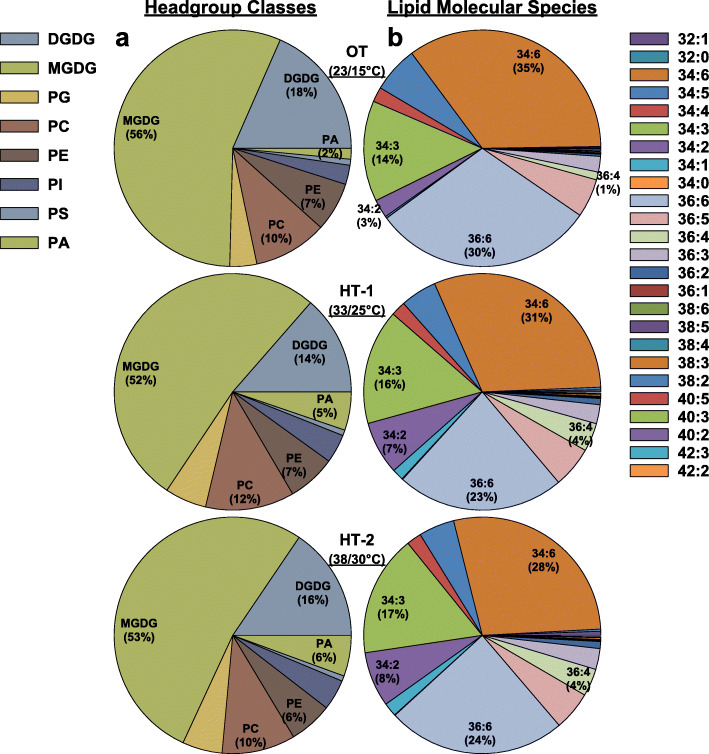
Pie charts depicting composition of various lipid head-group classes (**a**) and lipid molecular species (**b**) in Avanza 641 leaves under different temperature treatments. OT, optimal day/night temperatures; HT-1, high temperature treatment-1; HT-2, high temperature treatment-2. DGDG, digalactosyldiacylglycerol; MGDG, monogalactosyldiacylglycerol; PA, phosphatidic acid; PC, phosphatidylcholine; PE, phosphatidylethanolamine; PG, phosphatidylglycerol; PI, phosphatidylinositol; PS, phosphatidylserine. Lipid molecular species are identified as total acyl carbons: total double bonds

The composition of the leaf lipidome in terms of head-group classes for the control treatment (OT; 23/15°C) was dominated by three classes: MGDG (56 %), DGDG (18 %), and PC (10 %), which together accounted for ~ 85 % of the normalized mass spectral intensity (Fig. 2a). The same three head group classes in OT were also the predominant classes under HT-1 and HT-2. Together, they accounted for ~ 78 % of the total lipids in both high-temperature treatments (Fig. 2a).

When analyzed based on lipid molecular species composition, the leaf lipidome under OT was dominated by three lipid molecular species: 34:6 (35 %), 36:6 (30 %), and 34:3 (14 %), together accounting for 79 % of all lipid species (Fig. 2b). Under HT-1, the same three lipid species were dominant and together accounted for 70 % of the total lipid species (Fig. 2b). Similarly, the same three lipid species were dominant under HT-2 and together accounted for 69 % of the total lipids (Fig. 2b). The levels of 34:6 and 36:6 decreased by 11 and 23 %, respectively, under HT-1 compared to OT. Similarly, the levels of both lipid species decreased by 20 % under HT-2 compared to OT. In contrast, 34:3 increased by 14 and 21 % under HT-1 and HT-2, respectively. Substantial increases occurred under both HT treatments for 34:1 (556–680 %), 34:2 (133–167 %), and 36:4 (300 %) (Fig. 2b).

### Unsaturation levels and lipid species composition of head-group classes were modified under high temperature stress

**Fig. 3:**
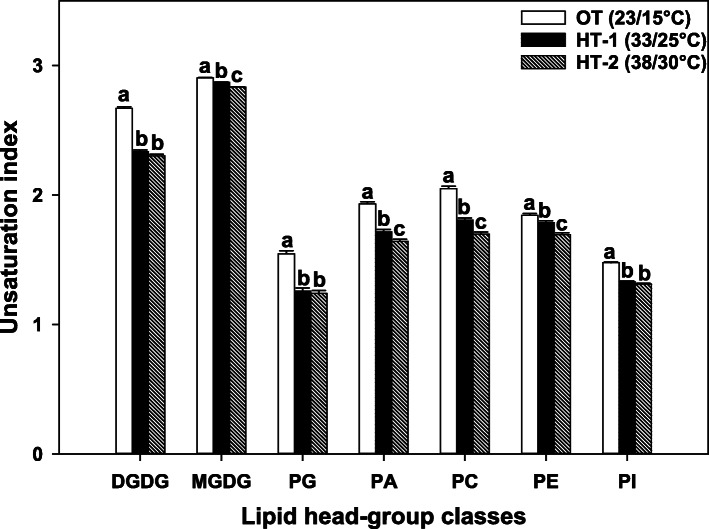
Changes in lipid unsaturation levels in response to high temperature stress. The unsaturation index for a lipid molecular species is the average number of double bonds per acyl chain, which is calculated by dividing the total number of double bonds in the acyl chains of that lipid molecular specie by its number of acyl chains. For a lipid head-group class, unsaturation index is calculated as previously described [29]: $\frac{\sum \left(\mathrm{unsaturation}\ \mathrm{in}\mathrm{dices}\ \mathrm{of}\ \mathrm{in}\mathrm{dividual}\ \mathrm{lipid}\ \mathrm{molecular}\ \mathrm{species}\ \mathrm{in}\ \mathrm{the}\ \mathrm{class}\times \mathrm{amount}\ \mathrm{of}\ \mathrm{each}\ \mathrm{species}\right)}{\sum \mathrm{amount}\ \mathrm{of}\ \mathrm{all}\ \mathrm{lipid}\ \mathrm{molecular}\ \mathrm{species}\ \mathrm{in}\ \mathrm{the}\ \mathrm{class}}$  Bars represent least-squares means, and error bars represent the corresponding standard errors. Least-squares means with different letters are significantly different according to Fisher’s least significant difference test at α = 0.05. OT, optimal day/night temperatures; HT-1, high temperature treatment-1; HT-2, high temperature treatment-2. DGDG, digalactosyldiacylglycerol; MGDG, monogalactosyldiacylglycerol; PA, phosphatidic acid; PC, phosphatidylcholine; PE, phosphatidylethanolamine; PG, phosphatidylglycerol; PI, phosphatidylinositol; PS, phosphatidylserine

The differences in the leaf lipidome between high temperature stress and OT were clearly reflected in the separation of the temperature treatments when subjected to principal component analysis, where principal components 1 and 2 captured 64.5 and 23.5 %, respectively, of the total variation in the lipid data (Supplementary Fig. S1, Additional file 2). High temperature stress resulted in a significant reduction of unsaturation levels across all head-group classes (Fig. 3). Furthermore, four head-group classes (MGDG, PA, PC, and PE) underwent a significant additional decrease in their unsaturation levels from HT-1 to HT-2.

**Fig. 4:**
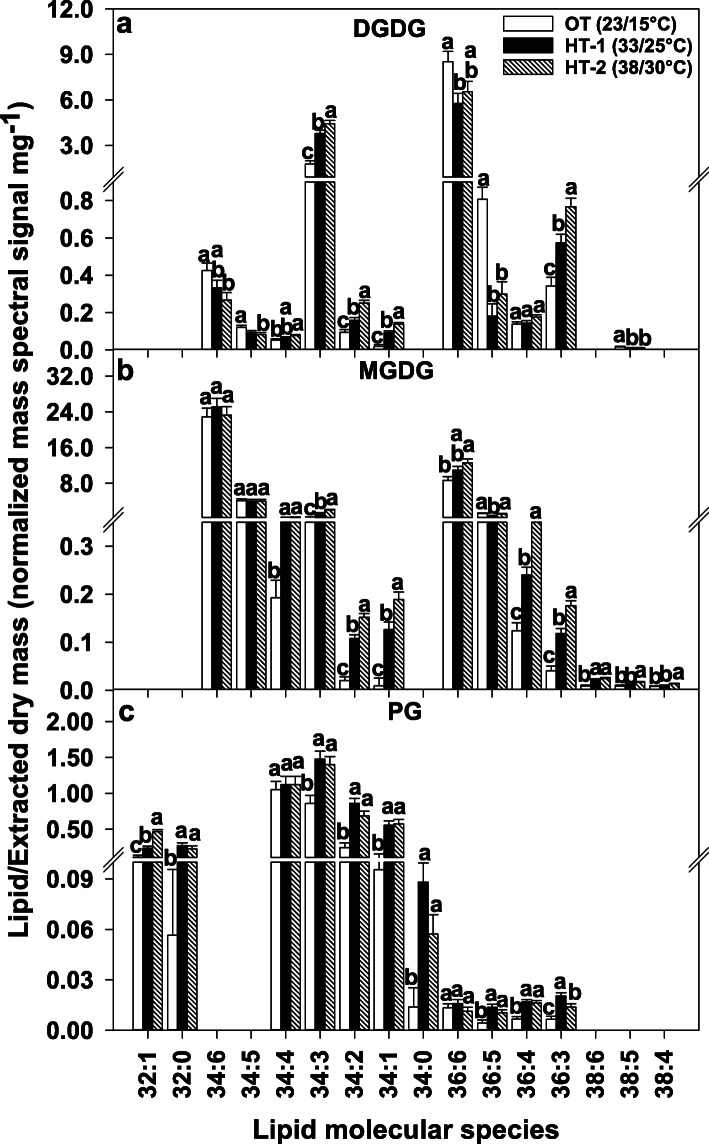
Changes in the levels of plastidic lipid molecular species in response to high temperature stress. All lipid molecular species shown passed both the limit of detection (LOD > 0.002 nmol) and the coefficient of variation (CoV < 0.3) cutoffs (details in “Methods”). Bars represent least-squares means and error bars represent the corresponding standard errors. Least-squares means with different letters are significantly different according to Fisher’s least significant difference test at α = 0.05. OT, optimal day/night temperatures; HT-1, high temperature treatment-1; HT-2, high temperature treatment-2; DGDG, digalactosyldiacyglycerol; MGDG, monogalactosyldiacylglycerol; PG, phosphatidylglycerol. Lipid molecular species are identified as total acyl carbons: total double bonds

**Fig. 5:**
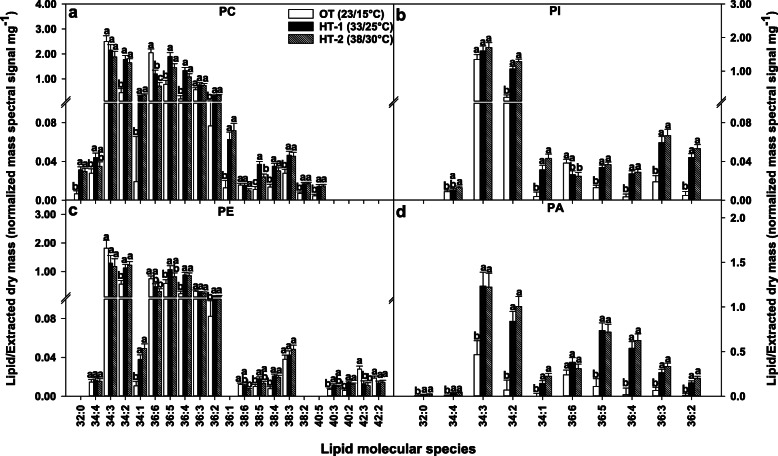
Changes in the levels of extraplastidic and phosphatidic acid (PA) lipid molecular species in response to high temperature stress. All lipid molecular species shown passed both the limit of detection (LOD>0.002 nmol) and the coefficient of variation (CoV<0.3) cutoffs (details in “Methods”). Bars represent least-squares means and error bars represent the corresponding standard errors. Least-squares means with different letters are significantly different according to Fisher’s least significant difference test at α = 0.05. OT, optimal day/night temperatures; HT-1, high temperature treatment- 1; HT-2, high temperature treatment-2; PC, phosphatidylcholine; PI, phosphatidylinositol; PE, phosphatidylethanolamine. Lipid molecular species are identified as total acyl carbons: total double bonds

Similarities and differences existed among the head-group classes in how a decrease in unsaturation index was achieved under high-temperature stress. Highly unsaturated lipids, i.e., those that contain two trienoic acyl chains such as 34:6 (18:3/16:3) and 36:6 (18:3/18:3), were significantly reduced in multiple head-group classes under either HT treatments. These included the plastidic lipid species DGDG(34:6) and DGDG(36:6) (Fig. 4), and the extraplastidic lipid species PC(36:6), PE(36:6), and PI(36:6) (Fig. 5). In contrast, the plastidic lipid species MGDG(36:6) significantly increased in response to HT, but no significant change occurred for MGDG(34:6), PG(36:6) (Fig. 4), and PA(36:6) (Fig. 5). Furthermore, several less unsaturated and saturated lipids significantly increased in response to HT. In the plastid, the lipid species DGDG 34:2, 34:1, and 36:3; MGDG 34:2, 34:1, 36:4, and 36:3; and PG 32:1, 32:0, 34:2, 34:1, 34:0, 36:4, and 36:3 increased (Fig. 4). Likewise, the extraplastidic lipid species PC 32:0, 34:2, 34:1, 36:4, 36:2, and 36:1; PE 34:2, 34:1, 36:4, and 36:2; and PI 34:2, 34:1, 36:4, 36:3, and 36:2 and PA 32:0, 34:2, 34:1, 36:4, 36:3, and 36:2 also increased (Fig. 5). When the fatty-acid compositions of the different lipid headgroup classes were examined, the same trend was observed, where saturated (16:0 and 18:0) and/or less unsaturated (18:1 and 18:2) acyl chains increased under high temperatures, whereas the highly unsaturated 18:3 acyl chain decreased (Table 1). Taken together, lipid remodeling at the lipid molecular species composition level contributed greatly to lowering unsaturation levels.

**Table 1:** Fatty-acid composition of various lipid headgroup classes under each temperature treatment

Lipid Headgroup Class	Acyl Chain	Amount/extracted dry mass (normalized mass spectral signal mg^**-1**^)		
		Optimal Temperature (23/15°C)^a^	High Temperature-1 (33/25°C)	High Temperature-2 (38/30°C)
DGDG	16:0	1.787±0.2152^c^	3.882±0.2152^b^	4.567±0.2152^a^
	16:3	0.4251±0.0404^a^	0.3327±0.0404^ab^	0.2674±0.0404^b^
	18:1	0.0203±0.0089^c^	0.0935±0.0089^b^	0.1383±0.0089^a^
	18:2	0.8069±0.0653^a^	0.1819±0.0653^b^	0.2997±0.0653^b^
	18:3	20.06±1.599^a^	15.82±1.599^a^	18.07±1.599^a^
MGDG	16:0	0.3512±0.1176^c^	1.299±0.1176^b^	2.008±0.1176^a^
	16:3	22.88±1.924^a^	25.11±1.924^a^	23.25±1.924^a^
	18:2	1.226±0.1269^a^	0.6864±0.1269^b^	1.133±0.1269^a^
	18:3	41.78±3.507^a^	48.98±3.507^a^	51.58±3.507^a^
PG	16:0	0.3323±0.1488^b^	1.421±0.1488^a^	1.554±0.1488^a^
	16:1	1.160±0.1371^b^	1.357±0.1371^ab^	1.586±0.1371^a^
	18:0	0.0139±0.0114^b^	0.0882±0.0114^a^	0.0578±0.0114^a^
	18:1	0.0954±0.0614^b^	0.5550±0.0614^a^	0.5750±0.0614^a^
	18:2	0.0043±0.0017^b^	0.0137±0.0017^a^	0.0104±0.00173^a^
	18:3	1.081±0.1208^a^	1.165±0.1208^a^	1.153±0.1208^a^
PC	16:0	2.974±0.4223^b^	4.285±0.4223^a^	3.927±0.4223^ab^
	16:1	0.0278±0.0046^b^	0.0440±0.0046^a^	0.0349±0.0046^ab^
	18:0	0.0129±0.0076^b^	0.0627±0.0076^a^	0.0718±0.00756^a^
	18:1	0.0321±0.0540^b^	0.3673±0.0540^a^	0.4209±0.0540^a^
	18:2	1.222±0.3178^b^	3.664±0.3178^a^	3.090±0.3178^a^
	18:3	7.404±0.6542^a^	6.490±0.6542^ab^	4.779±0.6542^b^
	20:3	0.0156±0.0020^a^	0.0155±0.0020^a^	0.0098±0.0020^a^
PE	16:0	2.386±0.3928^a^	2.452±0.3928^a^	2.446±0.3928^a^
	16:1	0.0146±0.0025^a^	0.0164±0.0025^a^	0.0155±0.0025^a^
	18:1	0.0106±0.0047^b^	0.0379±0.0047^a^	0.0492±0.0047^a^
	18:2	1.168±0.2548^b^	2.222±0.2548^a^	2.070±0.2548^a^
	18:3	3.921±0.5817^a^	3.328±0.5817^a^	2.607±0.5817^a^
	20:3	0.0121±0.0015^a^	0.0108±0.0015^ab^	0.0065±0.0015^b^
	22:0	0.0059±0.0014^b^	0.0141±0.0014^a^	0.0122±0.0014^a^
	24:0	0.0187±0.0020^a^	0.0135±0.0020^a^	0.0144±0.0020^a^
PA	16:0	0.5393±0.2988^b^	2.253±0.2988^a^	2.499±0.2988^a^
	16:1	0.0083±0.0051^b^	0.0354±0.0051^a^	0.0399±0.0051^a^
	18:1	0.0016±0.0302^b^	0.1435±0.0302^a^	0.2240±0.0302^a^
	18:2	0.1778±0.1971^b^	1.576±0.1971^a^	1.723±0.1971^a^
	18:3	1.064±0.3434^b^	2.765±0.3434^a^	2.598±0.3434^a^
PI	16:0	1.570±0.2148^b^	2.704±0.2148^a^	3.031±0.2148^a^
	16:1	0.0088±0.0018^b^	0.0105±0.0018^ab^	0.0130±0.0018^a^
	18:0	0.0050±0.0040^b^	0.0441±0.0040^a^	0.0532±0.0040^a^
	18:1	0.0037±0.0047^b^	0.0313±0.0047^a^	0.0430±0.0047^a^
	18:2	0.2405±0.0793^b^	1.147±0.0793^a^	1.375±0.0793^a^
	18:3	1.442±0.1587^a^	1.701±0.1587^a^	1.803±0.1587^a^

Lipid molecular species for which the acyl-chain composition is unambiguous were used in this analysis. For DGDG, this included 34:6 (16:3/18:3), 34:3 (16:0/18:3), 34:1 (16:0/18:1), 36:6 (18:3/18:3), and 36:5 (18:3/18:2). For MGDG, 34:6 (16:3/18:3), 34:3 (16:0/18:3), 36:6 (18:3/18:3), and 36:5 (18:3/18:2). For PG, 32:1 (16:0/16:1), 32:0 (16:0/16:0), 34:4 (16:1/18:3), 34:1 (16:0/18:1), 34:0 (16:0/18:0), 36:6 (18:3/18:3), and 36:5 (18:3/18:2). For PC, 32:0 (16:0/16:0), 34:4 (16:1/18:3), 34:3 (16:0/18:3), 34:2 (16:0/18:2), 34:1 (16:0/18:1), 36:6 (18:3/18:3), 36:5 (18:3/18:2), 36:1 (18:0/18:1), and 38:6 (18:3/20:3). For PE, 34:4 (16:1/18:3), 34:3 (16:0/18:3), 34:2 (16:0/18:2), 34:1 (16:0/18:1), 36:6 (18:3/18:3), 36:5 (18:3/18:2), 38:6 (18:3/20:3), 40:2 (18:2/22:0), and 42:2 (18:2/24:0). For PA: 32:0 (16:0/16:0), 34:4 (16:1/18:3), 34:3 (16:0/18:3), 34:2 (16:0/18:2), 34:1 (16:0/18:1), 36:6 (18:3/18:3), and 36:5 (18:3/18:2). For PI: 34:4 (16:1/18:3), 34:3 (16:0/18:3), 34:2 (16:0/18:2), 34:1 (16:0/18:1), 36:6 (18:3/18:3), 36:5 (18:3/18:2), and 36:2 (18:2/18:0). Acyl-chain amounts are shown as least-squares means with standard error. For each acyl chain, least-squares means with different letters are significantly different according to Fisher’s least significant difference test at α = 0.05

*DGDG* digalactosyldiacylglycerol, *MGDG* monogalactosyldiacylglycerol, *PA* phosphatidic acid, *PC* phosphatidylcholine, *PE* phosphatidylethanolamine, *PG* phosphatidylglycerol, *PI* phosphatidylinositol

^a^day/night temperatures

The changes in the levels of 34:3 species under HT treatments demonstrated opposite trends in chloroplast structural lipids (DGDG, MGDG, and PG) and extraplastidic structural lipids (PC and PE). While 34:3 species of DGDG, MGDG, and PG increased under HT treatments (Fig. 4), the opposite occurred for 34:3 species of PC and PE (Fig. 5). Although present in both chloroplasts and extraplastidic membranes, PA(34:3) significantly increased under HT treatments (Fig. 5). Overall, there was an increase in C34 DGDG lipids, a decrease in C36 DGDG lipids, and increases in C34 and C36 PA lipids under high-temperature treatments (Supplementary Fig. S2, Additional file 2).

## Discussion

Temperature treatments significantly affected the growth traits of Avanza 641. The observed failure of reproductive induction and continuation of vegetative growth in *B*. *carinata* at HT-1 and HT-2 is similar to previous observations in upland (*Gossypium hirsutum* L. [40]) and Pima (*Gossypium barbadense* L. [41]) cotton species and *B. napus* L. [42, 43]. Plants grown under HT-2 were shorter than those under OT and HT-1 (Fig. 1a). The leaf area per plant was greater under HT-1 than under OT and HT-2 (Fig. 1b). The greater leaf area observed under moderately high temperature (HT-1) treatment was due to reproductive induction failure, which promoted vegetative growth. The lower leaf area observed at the very high-temperature treatment (HT-2) was due to smaller leaves (data not shown). Total dry matter production per plant (weight of leaves, stems, pods, and roots) significantly decreased under HT-1 and HT-2 (Fig. 1d) largely due to lack of pods under high temperature stress.

The galactolipids MGDG and DGDG made up the lion’s share of total leaf lipids (66–74 %; Fig. 1a) in Avanza 641 under all three temperature treatments (OT, HT-1, and HT-2). This is expected given that galactolipids are the primary membrane lipids of plastids, and there is an overwhelming abundance of plastids in photosynthetic tissues [44–47].

A major response of Avanza 641 to high temperatures was the decrease in the unsaturation levels of membrane lipids (Fig. 3), which is a cumulative effect of lipid remodeling. Reducing lipid unsaturation levels in cellular membranes enables plants to maintain optimal membrane fluidity in response to increases in growth temperature [27, 29, 30, 32, 33, 48]. Although a common response in plant lipidomes under high-temperature stress is reductions in unsaturation levels, the magnitude of those reductions is greater for the heat-tolerant genotypes than the heat-susceptible genotypes [27, 29, 30, 32, 33, 48].

Two mechanisms that Avanza 641 employed to reduce lipid unsaturation levels were: decreasing highly unsaturated lipid species (e.g., 34:6 and 36:6) and increasing less unsaturated and/or saturated lipid species (e.g., 32:0, 34:2, 34:1, 36:3, and 36:2) (Figs. 2b, 4 and 5). This combination was the most evident in DGDG, PC, PE, and PI head-group classes (Figs. 4 and 5). 34:6 and 36:6 lipids that decreased under high temperatures (Table 1), contain the highly unsaturated linolenic acid (18:3), two in 36:6, and one in 34:6. 34:6, lipids also contain a 16:3 fatty acid alongside the 18:3. Fatty acid desaturase (FAD) 3, FAD7, and FAD8 are the enzymes that catalyze the conversion of 18:2 fatty acids to 18:3 (FAD3 in the ER and FAD7/8 in the plastid). The conversion of 16:2 to 16:3 in plastids is also catalyzed by FAD7/8. Decreasing lipid unsaturation levels by lowering the amount of 18:3 and/or 16:3 fatty acids through reducing the expression of *FAD3*, *FAD7*, and/or *FAD8* genes is likely a heat-acclimation mechanism in plants [30, 32, 49, 50].

**Fig. 6:**
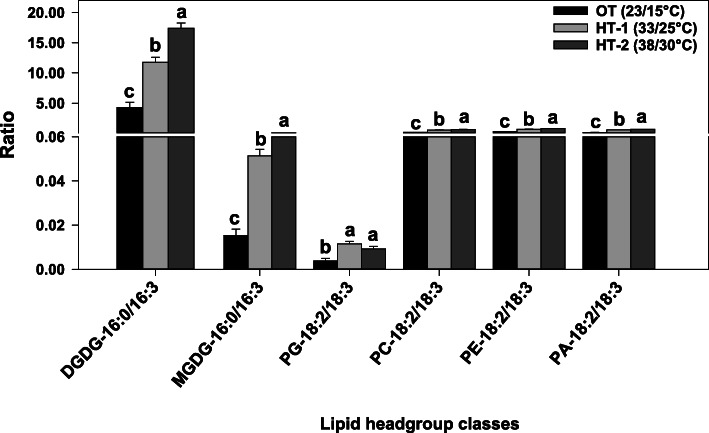
Changes in the ratios of 16:0/16:3 fatty acids in diagalactosyldiacylglycerol (DGDG) and monogalactosyldiacylglycerol (MGDG) and 18:2/18:3 fatty acids in phosphatidic acid (PA), phosphatidylcholine (PC), phosphatidylethanolamine (PE), and phosphatidylglycerol (PG) in respone to high temperatures. Bars represent least-squares means and error bars represent the corresponding standard errors. Least-squares means with different letters are significantly different according to Fisher’s least significant difference test at α = 0.05. OT, optimal day/night temperatres; HT-1, high temperature treatment- 1; HT-2, high temperature treatment-2. Acyl chains are identified as total acyl carbons: total double bonds

Many of the less unsaturated lipids that increased under high-temperature stress contained linoleic acid (18:2). Fatty acid desaturase 2 catalyzes the conversion of 18:1 fatty acids to 18:2 in the endoplasmic reticulum (ER). It has been reported that the expression of *FAD2* gene increased as an adaptation mechanism to heat stress in multiple species, including peanut (*Arachis hypogaea* L.) [32], soybean (*Glycine max* L. Merr.) [30], *Arabidopsis thaliana* L. [28], and *Atriplex lentiformis* L. [28]. In the present study, the ratio of 18:2/18:3 acyl chains in the extraplastidic structural lipids PC and PE significantly increased under high temperatures (Fig. 6) due to the increase in 18:2 levels and the decrese in 18:3 levels (Table 1). Thus, the reduced unsaturation levels of extraplastidic lipids in Avanza 641 due to the reduction in the amounts of lipids containing 18:3 fatty acid and the increase in the amounts of lipids containing 18:2 fatty acid might be a result of reduced expression of *FAD3* gene and increased expression of *FAD2* gene. Future studies can confirm this postulate by measuring the expression of *FAD3* and *FAD2* genes. It could also be possible that the decrease in membrane lipid unsaturation levels under high temperature stress is consistent with the rate of *de novo* fatty acid synthesis [49].

**Fig. 7:**
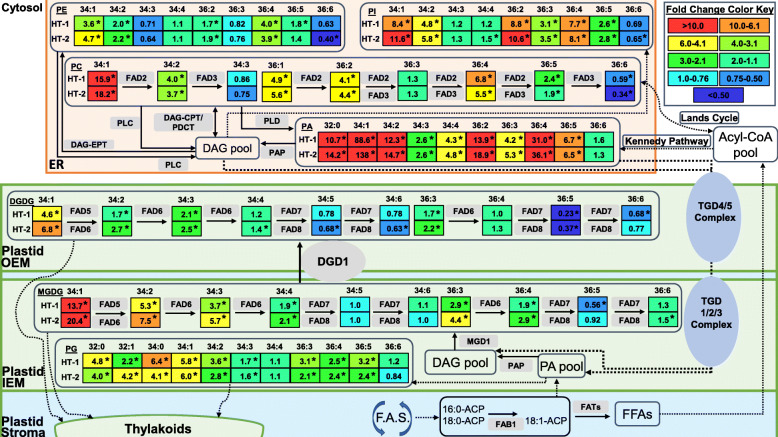
A lipid-metabolism map synthesizing the results of this study within the context of known lipid metabolic pathways [27, 45, 51–55]. Dashed arrows indicate either multi-step conversions or lipid trafficking pathways. Lipid molecular species are identified as total acyl carbons: total double bonds. The fold change values under each high temperature treatment were calculated as: HT-1 = (lipid value under HT-1/lipid value under OT) and HT-2 = (lipid value under HT-2/lipid value under OT). An asterisk (*) next to a fold-change value for a lipid indicates a significant change at α = 0.05 for that lipid under the corresponding treatment. ACP, acyl carrier protein; CoA, coenzyme A; DAG, diacylglycerol; DAG-CPT, CDP-choline:diacylglycerol cholinephosphotransferase; DAG-EPT, CDP-ethanolamine:diacylglycerol cholinephosphotranserase; ER, endoplasmic reticulum; FAB1, fatty acid biosynthesis 1; FAD, fatty acid desaturase; F.A.S., fatty acid synthesis; FAT, fatty acyl-ACP thioesterase; FFAs, free fatty acids; IEM, plastid inner envelope membrane; MGDG, monogalactosyldiacylglycerol; OEM, plastid outer envelope membrane; PA, phosphatidic acid; PAP, phosphatidic acid phosphatase; PC, phosphatidylcholine; PDCT, phosphatidylcholine:diacylglycerol cholinephosphotransferase; PE, phosphatidylethanolamine; PG, phosphatidylglycerol; PI, phosphatidylinositol; PLC, phospholipase C; TGD, trigalactosyldiacylglycerol (lipid transporter protein). OT, optimal day/night temperatures, 23/15°C; HT-1, high temperature treatment-1, 33/25°C; HT-2, high temperature treatment-2, 38/30°C

MGDG (34:6) did not decrease under high temperature stress, while MGDG (36:6) significantly increased (Fig. 4). Still the unsaturation index of MGDG decreased due to significant increases in several less unsaturated MGDG species (Fig. 4), such as the 13.7 and 20.4-fold increases in the monounsaturated MGDG(34:1) under HT-1 and HT-2, respectively (Fig. 7). At the fatty acid composition level, both MGDG and DGDG had a significant increase in the saturated fatty-acid palmitic acid (16:0) under high temperatures (Table 1), which is an initial product in *de novo* fatty-acid biosynthesis [46]. Falcone et al. [49] observed in *A*. *thaliana* a significant increase in 16:0 and a significant decrease in 16:3 for leaf membrane lipids in response to high temperature, which contributed to the decrease in membrane lipid unsaturation levels. Strikingly, this increase in 16:0 in response to high temperature, an increase attributed by the authors to newly synthesized fatty acids, was constant even in lipid biosynthesis mutants such as *fad7 fad8* and *fad6*, where little to no 16:3 is produced [49]. Considering that 16:3 levels decreased in DGDG (Table 1), which requires MGDG as precursor, and that 16:0 levels signifincatly increased in both DGDG and MGDG (Table 1), the increased biosynthesis of new fatty acids along with the downregulation of *FAD7/8* genes might have contributed to the reduction in the unsaturation level of both galactolipids. This preferential accumulation of 16:0 over 16:3 is reflected in the significant increase in the 16:0/16:3 ratios of both DGDG and MGDG under high temperatures (Fig. 6).

Apart from the alterations in the activity of desaturase enzymes, another method by which plants modulate membrane unsaturation levels in response to temperature stress is through adjusting the flux of acyl lipid metabolism between chloroplast and ER in the cytosol, via channeling of DAG/PA moieties with specific acyl-chain compositions [28, 56, 57]. In plants, the assembly of glycerolipids occurs in both plastids (prokaryotic pathway) and the ER (eukaryotic pathway) [46]. The substrate specificity of plastid and ER lipid-metabolizing enzymes permits only C16 fatty acids at the *sn*-2 position of plastid-derived glycerolipids and only C18 fatty acids at the *sn*-2 position of ER-derived glycerolipids [46, 51, 58]. Since the ER-derived *sn-1* 16:0 is not a substrate of plastid desaturases, further desaturation of this acyl chain does not occur in lipids in plastids. Thus, the eukaryotic pathway can result in lipids with a lower level of unsaturation than the prokaryotic pathway, e.g., eukaryotic C34:3 (16:0/18:3) vs. prokaryotic C34:6 (18:3/16:3). Therefore, to reduce the potential of fatty acid desaturation in chloroplast under high-temperature conditions, plants may suppress the input of the prokaryotic pathway (e.g., decreased formation of C34:6) and enhance the channeling of eukaryotic lipids (e.g., C34:3) to the chloroplast [28]. Supporting this mechanism, Li et al. [28] found an increase in C34 MGDG and DGDG lipids that have ER-derived DAG/PA moieties along with decreases in 34:6 and 36:6 lipids of both head groups under high-temperature conditions in *A. thaliana* and *A. lentiformis*. The same trends were observed in rapeseed (*B. napus*; same genus as that of Avanza 641), except that MGDG(36:6) showed an increase, which would be expected if the ratio of eukaryotic to prokaryotic lipid assembly was increased [57]. In the present study, it appears that Avanza 641 employed similar lipid channeling mechanism to remodel its lipidome in response to high temperature stress, and this mechanism was most evident for DGDG where 34:6 and 36:6 lipids significantly decreased and 34:3 significantly increased (Fig. 4). Furthermore, less unsaturated DGDG species that likely contain a C18 at the *sn*-2 position also increased (e.g., 34:2, 34:1, and 36:3) (Fig. 4).

We observed that although not statistically significant, both PC(34:3) and PE(34:3) decreased under high temperatures, whereas PA(34:3) significantly increased (Fig. 5). Relating these observations with an increase in C34 DGDG lipids, decrease in C36 counterparts, and increase in C34 and C36 PA lipids under high temperatures (Supplementary Fig. S2, Additional file 2) suggest a temperature-induced lipid pathway adjustments in plants. It could be that the C34:3 lipids from the eukaryotic pathway entering DGDG are derived ultimately from PC, with PA serving as a mode of transportation to shuttle the glycerolipid moiety into the chloroplast lipids. Li et al. [28] also observed the same C34 increase in PA that we observed and confirmed that the C34 glycerolipid moiety entering MGDG and DGDG through PA has a composition of 16:0/C18 (*sn*-1/*sn*-2), which significantly contributed to reducing the unsaturation levels of both head-groups. Thus, further studies can confirm whether the same glycerolipid moieties are getting channeled into the chloroplasts of Avanza 641 for lowering unsaturation levels of chloroplast membrane lipids.

The metabolic pathways that might have led to the alterations in the leaf lipidome of *B. carinata* in response to high-temperature stress are summarized in the following text within the broader context of plant lipid metabolism and are presented in Fig. 7. Briefly, fatty acids are synthesized in plastids where they can either become incorporated into plastidic lipids or exported to the ER and incorporated into extraplastidic lipids. In the ER, fatty acids attached to PC can return to the acyl-CoA pool via the Lands cycle; be transferred to DAG lipids by shuffling of acyl groups by CDP-choline:diacylglycerol choline phosphotransferase (DAG-CPT) or phosphatidylcholine:diacylglycerol cholinephosphotransferase (PDCT), removal of the entire phosphoryl head group via phospholipase C (PLC), removal of the PC head-group by phospholipase D (PLD) producing PA followed by removal of the remaining phosphate by phosphatidic acid phosphatase (PAP), or incorporation of modified fatty acids from the acyl-CoA pool into DAG through the Kennedy pathway; or be transferred to PE lipids by first converting to DAG lipids as previously mentioned followed by transfer of an ethanolamine head-group by CDP-ethanolamine:diacylglycerol cholinephosphotranserase (DAG-EPT) [59, 60]. Multiprotein transporter complexes consisting of trigalactosyldiacylglycerol (TGD) proteins 1 through 5 transport the ER-assembled lipids into the outer envelope membrane (OEM) (TGD4/5) and inner envelope membrane (IEM) (TGD1/2/3) of plastids in a unidirectional manner [61–69]. The ER-assembled PA translocated to the plastid membranes via the TGD complex can be dephosphorylated by phosphatidic acid phosphatase (PAP) to yield DAG, which is a precursor for galactolipids [52, 70]. Both eukaryotic and prokaryotic DAG species are galactosylated to form MGDG by monogalactosyldiacylglycerol synthase 1 (MGD1) localized at the IEM of plastid. MGDG could then be converted to DGDG by digalactosyldiacylglycerol synthase 1 (DGD1) [71]. Fatty acids esterified to glycerolipids in the plastid and ER can undergo desaturation by desaturases (FADs). Plants can modulate the level of unsaturation of membrane lipids by adjusting the activity of FADs. Under high-temperature stress, plants may respond by decreasing the activity of FAD3, FAD7, and/or FAD8 enzymes that catalyze the formation of the highly unsaturated linolenic (18:3) acid and/or increasing the activity of FAD2 and/or fatty acid biosynthesis enzymes that catalyze the formation of the less unsaturated linoleic (18:2) acid or the saturated palmitic acid (16:0), respectively. Plants can also suppress their prokaryotic pathway while enhancing their eukaryotic pathway. This promotes the channeling of less unsaturated ER-derived glycerolipids as a relatively quick means to lower unsaturation levels of chloroplast membrane lipids. Both lipid remodeling traits were observed in Avanza 641 (Fig. 7).

## Conclusions

High-temperature treatments (33/25 and 38/30°C) significantly affected the growth and development of Avanza 641 and caused alterations in the leaf lipidome. The lipid alterations appear to be acclimation mechanisms to maintain optimal membrane fluidity under high-temperature conditions. The remodeling of leaf lipidome was primarily manifested in terms of decreases in unsaturation levels of membrane lipids. The possible mechanisms that led to reductions in the lipid unsaturation levels were: (1) decreases in lipids containing highly unsaturated 18:3 fatty acids; (2) increases in lipids containing less unsaturated fatty acids such as 18:2 and 18:1 and/or saturated fatty acids such as 16:0; and (3) increase in the channeling of specific eukaryotic pathway-derived lipids to the chloroplast.

With the recent availability of *B. carinata* genotypes with varying levels of thermotolerance, it is now possible to compare within the species the magnitude of acclimation changes in their lipidomes and related enzyme activities to identify lipid-related biomarkers for heat tolerance. Further studies that compare the lipidomes of different tissue types or the changes in the lipidome under different spatio-temporal environments would provide additional insight necessary for improving carinata’s heat tolerance. Lastly, comparative studies that leverage the existing body of work in other *Brassica* species such as canola or rapeseed (*B.napus* L.) could potentially identify stable biomarkers applicable to the entire genus.

## Methods

### Plant material and growth conditions

The plant material used in this study was a released variety of *B. carinata*, named Avanza 641 (seeds were obtained from Dr. Ramdeo Seepaul at the University of Florida; original seed source was Agrisoma Biosiences Inc., Quebec, Canada; no permissions were necessary to collect seed samples). It is a current commercial cultivar (not transgenic) in the southeastern United States. This cultivar is classified as heat-sensitive in early-season vegetative growth and moderately heat-sensitive during the seed germination stage using seed vitality traits [25].

All plant experiments were performed in accordance with relevant guidelines and regulations. Plants were grown under sunlit-controlled environmental conditions known as the Soil-Plant-Atmosphere-Research (SPAR) chambers at Mississippi State University, Starkville, MS [72]. Each SPAR chamber has a soil bin (1 m deep x 2 m long x 0.5 m wide) to hold the belowground parts of the plants and a Plexiglas chamber (2.5 m tall x 2 m long x 1.5 m wide) for the aboveground parts. The Plexiglas permits 97 % of the visible solar radiation and does not discriminate within the visible light spectrum (400–700 nm). Daylength in the SPAR chambers for the duration of the study varied from 12.56 to 14.17 h.

Seeds of Avanza 641 were sown on 7 June 2018 in polyvinyl chloride (PVC) pots (15.24 cm diameter, 30.48 cm height, and 5.5 L volume) filled with a 3:1 (v/v) soil: sand mixture. Pots were filled with pure sand (particle size less than 0.3 mm) with 500 g of gravel at the bottom of each pot, inserted into the soil bin filled with sand so that the top of the pot aligns with the top portion of the soil bin. Five seeds were sown in each pot, but thinning was performed one week after seedling emergence to maintain only one plant per pot. Thus, nine pots (replications), each containing one plant, were maintained in each of the three SPAR chambers used in the study. The plants/pots were arranged in three rows with 0.66-m row spacing and nine plants m^− 2^.

To ensure that the plants did not experience any water stress, pots were irrigated with full-strength Hoagland nutrient solution [73] through an automated drip irrigation system, one dripper per pot with each dripper emitting 50 ml min^− 1^. The amount of irrigation delivered was 120 % of the measured previous day’s evapotranspiration [72], split into three times during the day. Seedlings in all SPAR chambers were initially grown under optimal temperatures (OT; 23/15°C day max/night min) until 8 days after sowing. After that, one of the three temperature treatments [OT, high-temperature treatment-1 (HT-1; 33/25°C), and high-temperature treatment-2 (HT-2; 38/30°C)] were randomly assigned to each of the three SPAR chambers. The optimal and high-temperature regimes were chosen based on Persaud [25] and Boote et al. [74]. Persaud [25] found that 22/14°C serves as optimal temperature for carinata during early season growth and development and significant heat damage will start at 27/19°C. Boote et al. [74] reported the cardinal temperatures for various growth and development processes of carinata; the optimal temperatures were generally around 25 °C and ceiling temperatures were generally ≥ 35 °C. Air temperature in each SPAR unit was monitored and adjusted every 10 s throughout the day and night and maintained within set points ± 0.5 °C by passing conditioned air through the plant canopy with sufficient velocity to cause leaf flutter (4.7 km h^− 1^) and was returned to the air-handling unit just above the soil level. Chilled ethylene glycol was supplied to the cooling system via several parallel solenoid valves that opened or closed depending on the cooling requirement. To fine-tune the air temperature, two electrical resistance heaters provided short pulses of heat as needed. The CO_2_ concentration [CO_2_] in each SPAR unit was monitored and adjusted every 10 s throughout the day and maintained at 420 ± 10 ppm using a dedicated LI-6250 CO_2_ analyzer (LI-COR, Inc., Lincoln, NE). The chamber air temperature, [CO_2_], and pot watering in each SPAR unit as well as continuous monitoring of environmental and plant gas exchange variables were controlled by a dedicated computer system [72]. The daytime temperature was initiated at sunrise and returned to the night-time temperature 1 h after sunset. The temperature treatment period lasted until the final harvest on 29 August 2018 (84 days after sowing).

The relative humidity of each chamber was monitored with a sensor (HMV 70Y, Vaisala, Inc., San Jose, CA) installed in the returning path of airline ducts. The mean day and night vapor pressure deficits in the units estimated from these measurements as per Murray [75] were 0.65 ± 0.01 kPa at OT, 0.97 ± 0.04 kPa at HT-1, and 1.12 ± 0.05 kPa at HT-2 during temperature treatment period.

Plants were grown under ambient light conditions. During the experiment, incoming daily solar radiation (285–2800 nm) outside of the SPAR units was measured with a pyranometer (Model 4–8; The Eppley Laboratory Inc., Newport, RI) and ranged from 4.40 to 34.25 MJ m^− 2^ d^− 1^ with an average of 22.54 ± 14.40 MJ m^− 2^ d^− 1^. Variable density shade cloths, designed to simulate canopy spectral properties and placed around the edges of the plant canopy, were adjusted regularly to match canopy height and to eliminate the need for border plants.

### Measurement of growth traits

Plant height was measured as the distance between the soil surface and the apical meristem at the final harvest (84 days after sowing). The number of pods per plant was counted on all plants at the final harvest. The whole plant leaf area was measured using a LI-3100 leaf-area meter (LI-COR, Inc., Lincoln, NE) at the time of final harvest. Only green and yellow leaves were used for leaf area measurement. Dried leaves were not used for leaf area measurements but were saved for measuring leaf dry weight. At harvest, the soil medium with a root system from each pot was placed on fine mesh screens and washed thoroughly using a gentle stream of water to collect roots. Plant component parts, leaves, stems, pods and roots, were oven-dried at 75 °C until a constant weight was achieved. All growth traits were measured on nine plants (replications) per temperature treatment.

### Leaf lipid extraction

Leaf discs (~ 0.5 g) were collected at 74 days after sowing (i.e., on the 66th day of the temperature treatment period) between the hours of 10:00 and 11:00. Leaf discs were collected from the 3rd fully mature leaf from the top from five plants (replications) per temperature treatment. Leaf disc collection and lipid extraction were carried out as previously described [76]. Briefly, ten leaf discs per plant were quickly acquired using a no. 3 cork borer and transferred immediately to 6 ml of pre-heated (75 °C for 15 min) isopropanol containing 0.01 % butylated hydroxytoluene (BHT) in a 50- ml glass tube. After adding leaf discs, the tubes were again kept at 75 °C for 15 min to deactivate lipid-hydrolyzing enzymes. The solvent with leaf discs was cooled to room temperature, 3 ml of chloroform and 1.2 ml of distilled water were added to the solvent, and the sample tubes were stored at -20 °C until further processing.

Further lipid extraction was carried out by shaking the leaf discs on an orbital shaker for 1 h at room temperature. The solvent in each tube was transferred with a Pasteur pipette to a new tube, leaving behind the leaf discs. To the leaf discs, 4 ml of chloroform: methanol (2:1) with 0.01 % BHT was added and left on an orbital shaker overnight at room temperature. The following day, the solvent in each tube was transferred with a Pasteur pipette to the tube that contained the initial solvent transferred on the previous day. The three steps: addition of 4 ml chloroform: methanol (2:1) with 0.01 % BHT, shaking overnight at room temperature, and transfer of the solvent were repeated four more times until the leaf discs appeared white. A nitrogen evaporator was used to evaporate the solvent accumulated from the previous steps. Then, the dried lipid extract in each tube was dissolved in 1 ml of chloroform and transferred to a 2-ml clear glass vial with a Teflon-lined screw cap, which was stored at -80 °C until ready for shipment to the Kansas Lipidomics Research Center. Samples were prepared for shipment by evaporating the chloroform. Thereafter, the samples were packed with bubble wrap for protection and shipped with dry ice. Leaf discs were oven-dried at 105 °C overnight, cooled to room temperature, and weighed to five decimal places to express lipid content on a dry weight basis.

### Lipidomics analysis conducted with a triple quadrupole mass spectrometer

Lipid extracts were prepared in chloroform with appropriate internal standards. Electrospray ionization-triple quadrupole mass spectrometry (Applied Biosystems API 4000) was used to detect lipids [29, 77]. Lipid molecular species were identified using either precursor or neutral loss scanning and quantified using internal standards for each head-group class. A quality-control (QC) pool was created by combining aliquots from all samples. Identical samples made from this QC pool were analyzed recurrently among the experimental samples, which allowed calculating the coefficient of variation (CoV) for each lipid analyte. Lipid levels were quantified as normalized intensity per milligrams of leaf dry weight, where a value of one is the intensity of 1 nmol of internal standard. A correction factor of 2.8 was applied to the galactolipids to correct the higher response of unsaturated lipids than the saturated internal standards. Lipid analytes that were either below the limit of detection (intensity corresponding to 0.002 nmol) or had a CoV value greater than 0.3 were excluded from further analysis.

### Unsaturation index calculation

Lipids with fatty acid chains containing double bonds are considered unsaturated, and the unsaturation index can represent their degree of unsaturation. This index for a lipid molecular species is the average number of double bonds per acyl chain, which is calculated as followed:

1 $$\frac{\text{Totalnumberofdoublebondsintheacylchainsofthelipidmolecularspecies}}{\text{Numberofacylchainsinthelipidmolecularspecies}}$$

For a lipid head-group class, the unsaturation index was calculated as previously described [29]:

2 $$ < mathdollar>\frac{\sum \left(\mathrm{unsaturation}\ \mathrm{in}\mathrm{dices}\ \mathrm{of}\ \mathrm{in}\mathrm{dividual}\ \mathrm{lipid}\ \mathrm{molecular}\ \mathrm{species}\ \mathrm{in}\ \mathrm{the}\ \mathrm{class}\times \mathrm{amount}\ \mathrm{of}\ \mathrm{each}\ \mathrm{species}\right)}{\sum \mathrm{amount}\ \mathrm{of}\ \mathrm{all}\ \mathrm{lipid}\ \mathrm{molecular}\ \mathrm{species}\ \mathrm{in}\ \mathrm{the}\ \mathrm{class}} $$

### Data analysis

The experimental design was a completely randomized design. The GLIMMIX procedure in SAS (Version 9.4, SAS Institute) was used to analyze variance and estimate least-squares means and standard errors. Replication was considered as a RANDOM effect. The LSMEANS option in the GLIMMIX procedure was used to separate least-squares means based on Fisher’s least significant difference (LSD) test at α = 0.05. Principal component analysis (PCA) was conducted in MetaboAnalyst 5.0 (metaboanalyst.ca) [78, 79].

## Supplementary Information

**Additional file 1: Table S1.** Data on 105 lipid analytes as normalized intensity per milligrams of leaf dry weight. **Table S2.** Analysis of variance results on the effects of temperature treatments on lipid head-group unsaturation indices, lipid molecular species, and levels of head-group class sub-pools.

**Additional file 2: Figure S1.** Principal component analysis (PCA) scores plot revealing distinguishable lipid profiles among the temperature treatments. **Figure S2.**Changes in the levels of digalactosyldiacylglycerol (DGDG) and phosphatidic acid (PA) sub-pools in Avanza 641 leaves in response to high temperature stress.

## Data Availability

All lipid data generated or analyzed in this study are included in this published article and its Supplementary Table S1, Additional file 1.

## Abbreviations

DAG-CPT: CDP-choline:diacylglycerol cholinephosphotransferase
DAG-EPT: CDP-ethanolamine:diacylglycerol cholinephosphotranserase
DGD1: Digalactosyldiacylglycerol synthase 1
DGDG: Digalactosyldiacylglycerol
ER: Endoplasmic reticulum
ESI-MS/MS: Electrospray ionization-triple quadrupole mass spectrometry
FAD: Fatty acid desaturase
IEM: Inner envelope membrane
MGD1: Monogalactosyldiacylglycerol synthase 1
MGDG: Monogalactosyldiacylglycerol
OEM: Outer envelope membrane
PA: Phosphatidic acid
PAP: Phosphatidic acid phosphatase
PC: Phosphatidylcholine
PDCT: Phosphatidylcholine:diacylglycerol cholinephosphotransferase
PE: Phosphatidylethanolamine
PG: Phosphatidylglycerol
PI: Phosphatidylinositol
PLC: Phospholipase C
PLD: Phospholipase D
SPAR: Soil-plant-atmosphere-research chamber
TGD1-5: Trigalactosyldiacylglycerol proteins 1-5
