# Correction to: Alterations in the leaf lipidome of Brassica carinata under high-temperature stress

**DOI:** 10.1186/s12870-021-03210-3

**Published:** 2021-10-06

**Authors:** Zolian Zoong Lwe, Saroj Sah, Leelawatti Persaud, Jiaxu Li, Wei Gao, K. Raja Reddy, Sruthi Narayanan

**Affiliations:** 1 Department of Plant and Environmental Sciences, Clemson University, Clemson, SC 29634 USA; 2 Department of Biochemistry and Molecular Biophysics, Kansas State University, Manhattan, KS 66506 USA; 3 Department of Biochemistry, Molecular Biology, Entomology and Plant Pathology, Mississippi State University, Starkville, MS 39762 USA; 4 Plant and Soil Sciences, Mississippi State University, Starkville, MS 39762 USA; 5 USDA UVB Monitoring and Research Program, Natural Resource Ecology Laboratory, Department of Ecosystem Science and Sustainability, Colorado State University, Fort Collins, CO 80523 USA

**Correction to: BMC Plant Biol 21, 404 (2021)**

**https://doi.org/10.1186/s12870-021-03189-x**

Following publication of the original article [1], author has found a minor typesetting error in Fig. 6. The correct figure is given below:

**Fig. 6:**
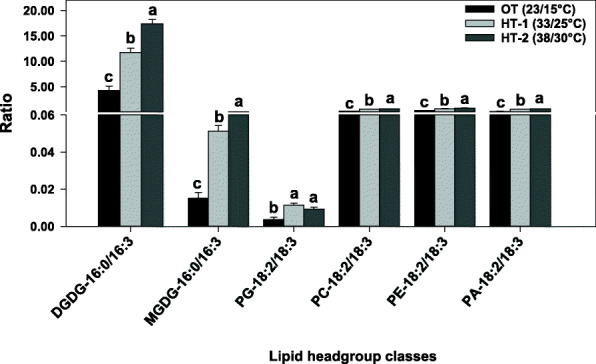
Changes in the ratios of 16:0/16:3 fatty acids in diagalactosyldiacylglycerol (DGDG) and monogalactosyldiacylglycerol (MGDG) and 18:2/18:3 fatty acids in phosphatidic acid (PA), phosphatidylcholine (PC), phosphatidylethanolamine (PE), and phosphatidylglycerol (PG) in response to high temperatures. Bars represent least-squares means and error bars represent the corresponding standard errors. Least-squares means with different letters are significantly different according to Fisher’s least significant difference test at α = 0.05. OT, optimal day/night temperatures; HT-1, high temperature treatment- 1; HT-2, high temperature treatment-2. Acyl chains are identified as total acyl carbons: total double bonds

The correction does not have any effect on the results or conclusions of the paper. The original article has been corrected.
