# Supplementary material for: Alterations in the leaf lipidome of Brassica carinata under high-temperature stress
Source: BMC Plant Biol. 2021 Sep 6;21:404. doi: 10.1186/s12870-021-03189-x (PMC8419912; doi:10.1186/s12870-021-03189-x)
Supplement: Supplementary file 2 — Additional file 2: Figure S1. Principal component analysis (PCA) scores plot revealing distinguishable lipid profiles among the temperature treatments. Figure S2.Changes in the levels of digalactosyldiacylglycerol (DGDG) and phosphatidic acid (PA) sub-pools in Avanza 641 leaves in response to high temperature stress. [file 12870_2021_3189_MOESM2_ESM.docx]

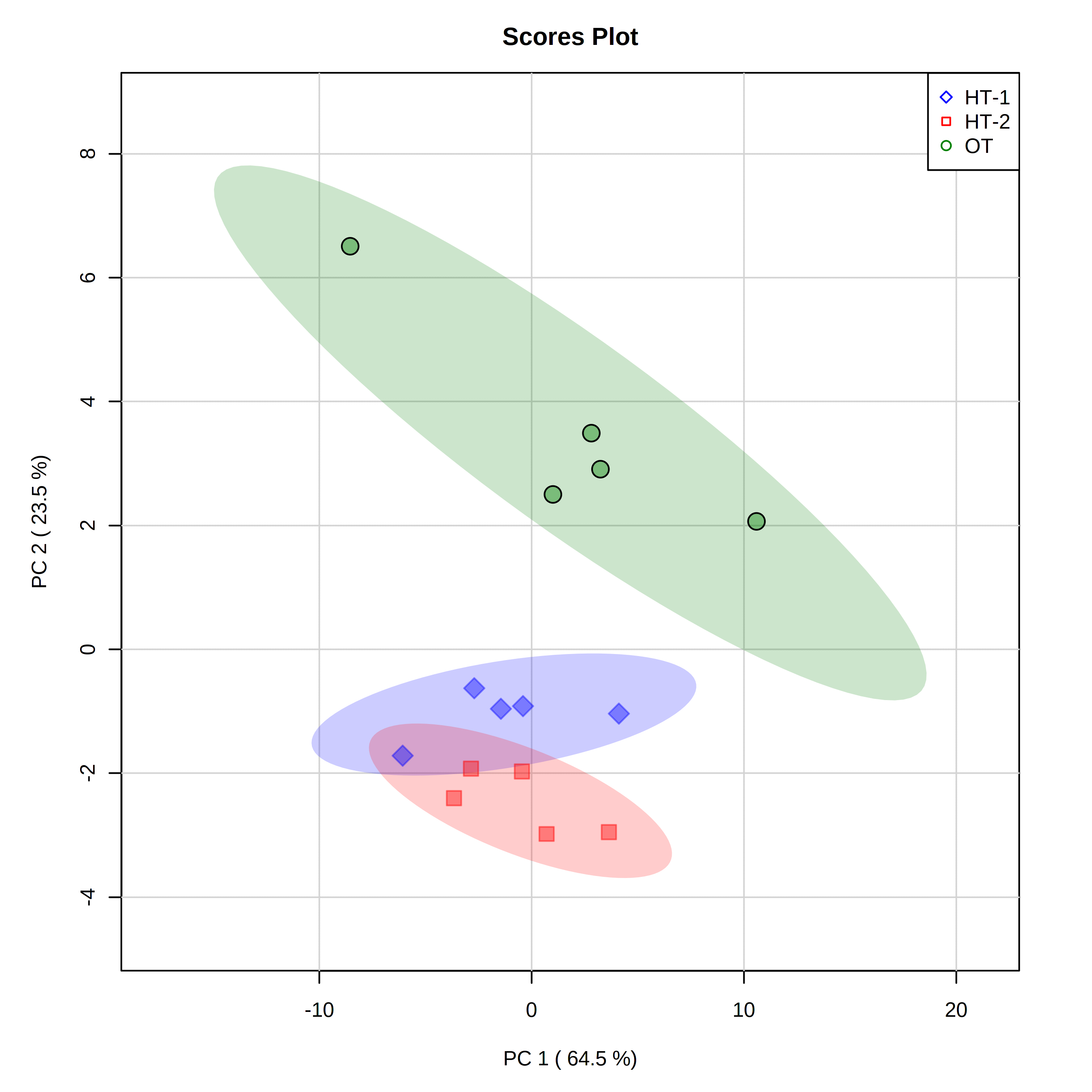


**Supplemental Figure S1.** Principal component analysis (PCA) scores plot revealing distinguishable lipid profiles among the temperature treatments. PCA was conducted in Metaboanalyst 5.0 (metaboanalyst.ca) using all 105 lipid analytes detected in all temperature treatments (Supplemental Table S1): optimal day/night temperatures (OT, 23/15°C), high-temperature treatment-1 (HT-1, 33/25°C), and high-temperature treatment-2 (HT-2, 38/30°C, red squares). Ellipses surrounding samples of each treatment represent 95% confidence regions. Five replicates were used per treatment. No data scaling or normalization was carried out before analysis.





**Supplemental Figure S2.** Changes in the levels of digalactosyldiacylglycerol (DGDG) and phosphatidic acid (PA) subpools in Avanza 641 leaves in response to high temperature stress. C34 includes *sn*1-C16/*sn*2-C18 and *sn*1-C18/*sn*2-C16 lipids. C36 includes C18/C18 lipids. Bars represent least-squares means, and error bars represent the corresponding standard errors. Least-squares means with different letters are significantly different according to Fisher’s least significant difference test at α = 0.05. OT, optimal day/night temperatures; HT-1, high-temperature treatment-1; HT-2, high-temperature treatment-2.
